# Qingluo Tongbi Formula Alleviates Hepatotoxicity Induced by *Tripterygium wilfordii* Hook. F. by Regulating Excessive Mitophagy Through the PERK-ATF4 Pathway

**DOI:** 10.3389/fphar.2022.918466

**Published:** 2022-07-07

**Authors:** Linluo Zhang, Jie Zhou, Zhe Feng, Baoping Jiang, Changqing Li, Lingling Zhou, Xueping Zhou

**Affiliations:** ^1^ The First Clinical Medical College, Nanjing University of Chinese Medicine, Nanjing, China; ^2^ Jiangsu Provincial Key Laboratory of Pharmacology and Safety Evaluation of Material Medical, School of Pharmacy, Nanjing University of Chinese Medicine, Nanjing, China

**Keywords:** *Tripterygium wilfordii* hook. f*.*, hepatotoxicity, qingluo tongbi formula, mitophagy, ERS, PERK

## Abstract

Qingluo Tongbi Formula (QTF) is an empirical formula of Chinese medicine master Zhongying Zhou for the treatment of rheumatoid arthritis. Although including *Tripterygium wilfordii* Hook. F. (TW), it has not shown liver toxicity in clinical application for many years. Our previous studies have shown that QTF can significantly reduce TW-caused hepatotoxicity, but the mechanism is still unclear. This study aimed to explore the important roles of mitophagy and endoplasmic reticulum stress (ERS) and the relationship between them in QTF in alleviating TW-induced hepatotoxicity. *In vivo*, C57BL/6J female mice were used to build a model of TW-induced liver toxicity; Then mice were randomly divided into control, TW, TW + RG, TW + PN, TW + SA, TW + BM, and QTF groups. After intragastric administration for 7 days, the levels of alanine aminotransferase (ALT), aspartate aminotransferase (AST) and lactate dehydrogenase (LDH) in serum were detected; H and E staining, Oil Red O staining, transmission electron microscopy, Western blotting, and RT-qPCR were used to detect the pathological changes in liver tissue, the levels of ERS and mitophagy related proteins and genes, including GRP78, PERK, DRP1, LC3, etc., *In vitro*, triptolide (TP), catalpol (CAT), and panax notoginseng saponins (PNS), the main active ingredients of QTF, were selected. The mitophagy inhibitor, ERS inhibitor, and PERK inhibitor were used to further study the relationship between TW-induced ERS and mitophagy in HepaRG cells. The results showed that, QTF reduced excessive mitophagy and ERS in TW-induced hepatotoxicity in C57BL/6J mice, and the attenuating effects of RG and PN in QTF were most obvious, and they also significantly restrained the TW-induced ERS and mitophagy by the PERK-ATF4 pathway. Furthermore, PNS was superior to CAT in inhibiting the expression levels of GRP78, PERK, and ATF4, while CAT was superior to PNS in reversing the expression levels of DRP1, P62, and LC3. The combination of CAT and PNS had the best attenuating effect and the most significant regulation on ERS and mitophagy. In conclusion, QTF can alleviate TW-induced hepatotoxicity by differentially downregulating the PERK-ATF4 pathway and excessive mitophagy by different components.

## 1 Introduction

*Tripterygium Wilfordii* Hook. F. (TW) is commonly used in traditional Chinese medicine (TCM) to treat rheumatoid arthritis (RA), systemic lupus erythematosus (SLE), and various tumor diseases, because of its significant immunomodulatory, anti-inflammatory, and anti-tumor effects (Lv et al., 2019). However, the potential side effects of TW, such as hepatotoxicity and so on, seriously limit its further clinical application (Zhang et al., 2018). It is one of the current research hotspots to clarify the mechanism of the TW-induced hepatotoxicity and to reduce or/and eliminate its hepatotoxicity while ensuring the efficacy.

The application of TW in TCM has a long history, and the compound compatibility of TCM is one of the important methods to enhance the efficacy or/and reduce its liver toxicity. QTF is composed of TW, *Rehmannia glutinosa* (Gaertn.) DC. (RG), *Panax notoginseng* (Burkill) F. H. Chen (PN), *Sinomenium acutum* (Thunb.) Rehder and E. H. Wilson (SA), and *Bombyx mori L.* (BM). As an empirical prescription from Chinese medicine master Prof. Zhongying Zhou in the clinical treatment of RA with Yin Deficiency and Collateral Heat (Yinxu Luore) Syndrome, no liver toxicity of QTF was found after many years of application (Liu et al., 2015; Yang et al., 2020). Our team previous research has proved that QTF can reduce and eliminate the TW-induced liver toxicity in SD rats and HepaRG cells (Yu et al., 2022), but the specific molecular biological mechanism in QTF alleviating TW-induced hepatotoxicity is not yet very clear.

Mitochondria is a key organelle for aerobic respiration and energy metabolism, so its normal number and function are very important. Mitochondrial autophagy (also known as mitophagy) maintains the homeostasis and balance of mitochondria by removing damaged mitochondria to regulate cell fate. At the same time, the endoplasmic reticulum (ER) and mitochondria are closely connected and interact by the mitochondrial-related endoplasmmic reticulum membranes (MAMs), coordinately regulating autophagy and apoptosis. Our previous study showed that ER stress (ERS) and excessive autophagy, which are the important mechanisms of TP-induced hepatocyte injury in HepaRG cells (Yu et al., 2022; Zhang et al., 2022). However, the role that mitophagy plays in reducing the TW-induced hepatotoxicity by QTF and the relationship between ERS and mitophagy are not very clear and need to be further studied.

This study focused on investigating the role of mitophagy in QTF in alleviating TW-induced liver injury, and the relationship between ERS and mitophagy, to elucidate the specific mechanism in alleviating the TW-induced hepatotoxicity by QTF.

## 2 Materials and Methods

### 2.1 *In vivo* Study

#### 2.1.1 Preparation of Qingluo Tongbi Formula

The Chinese herbal medicines of QTF were purchased from the Bozhou Medicinal Material Center (batch number: 190816, Anhui, China), and were identified by the School of Pharmacy at Nanjing University of CM (NJUCM, 20180922-20180926, Nanjing, China). The QTF is composed of TW, RG, PN, SA, and BM by the ratio of 15:15:3:15:10. The formulas were decocted with 11 times pure water for 1.5 h, and then decocted with 7 times pure water for 1.5 h, and the two were mixed, concentrated, and filtered. The crude drug contents of TW, TW + RG (the ratio is 1:1), TW + PN (the ratio is 5:1), TW + SA (the ratio is 1:1), TW + BM (the ratio is 3:2), and QTF were 1.95, 3.90, 2.34, 3.90, 3.25, and 7.54 g/ml, respectively.

In order to ensure the consistency and reproducibility of QTF extracts, the content of the main active components was analyzed by the HPLC method. Samples were separated on the Agilent ZORBAX SB-C18 column (4.6 × 250 mm, 5 μm), maintained at 30°C. The mobile phase consisted of acetonitrile (A), 0.1% phosphoric acid and 0.05% triethylamine (B) in a gradient elution: 5% A (0–2 min), 5%–10% A (2–10 min), 10% A (10–12 min), 10%–12% A (12–15 min), 12%–30% A (15–35 min), and 30% A (35–40 min). The flow rate was 0.8 ml/min, the injection volume was 10 μl, and the detection wavelength was set at 203 nm. The HPLC chromatogram and quantitative data of the main chemical components are used as supplementary material (Supplementary Figure S1).

#### 2.1.2 Animals and Treatment

C57BL/6J female mice (6–8 weeks old, 18–22 g) were purchased from Hangzhou Medical College [Animal license number: SCXK (Zhe)2019-0002], and were treated in the Animal Center of Nanjing University of CM with the temperature of 23 ± 2°C, humidity 40%–60%, and a standard 12 h light/12 h dark cycle, fed standard pelleted diet and provided free drinking water.

First, different doses of TW decoction (containing 0.4875, 0.975, and 1.95 g/ml of TW, respectively) were administered orally to establish an ideal mouse model of liver injury. Then, mice were divided into 7 groups, with 8 mice in each group to study the attenuation mechanism, namely: control, TW, TW + RG, TW + PN, TW + SA, TW + BM, and QTF groups. The mice were sacrificed after 7 days of oral administration at a dose of 0.02 ml/g body weight, and the serum was extracted to detect alanine aminotransferase (ALT), aspartate aminotransferase (AST) and lactate dehydrogenase (LDH). The liver tissue was detected by H and E and Oil Red O staining, and the protein and RNA were extracted for Western blotting and RT-qPCR. All animal experiments were approved by the Laboratory Animal Center of Nanjing University of CM and carried out in accordance with the Principles of Animal Use and Guidelines for the Care and Use of Laboratory Animals.

#### 2.1.3 Hematoxylin-Eosin Staining of Liver Tissue

The fresh liver tissue was fixed in 4% paraformaldehyde solution, paraffin-embedded, sectioned, and then stained with H and E. Histopathological scores were scored as previously described (Brunt 2000; Ramos et al., 2015).

#### 2.1.4 Oil Red O Staining of Liver Tissue

The fresh liver tissue was fixed with 4% paraformaldehyde, stained with Oil Red O working solution for 10 min, and then destained with 60% isopropanol. Rinse three times with distilled water, observe and photograph under the microscope. Oil Red O quantitative statistical analysis was performed using Image-Pro Plus 6.0 software (Media Cybernetics, Inc., Rockville, MD, United States).

Fat percentage (%) = fat area/tissue area × 100%

#### 2.1.5 Transmission Electron Microscope Observation of Liver Tissue

Fresh mouse liver tissue was fixed in TEM fixative, dehydrated, infiltrated, embedded, sliced, photographed and analyzed under TEM.

#### 2.1.6 Detection of Liver Functions

After mice were treated, blood was collected from the orbit, left at room temperature for 4 h, centrifuged at 3,000 rpm/min at 4°C for 10 min, the supernatant was taken, and the levels of ALT, AST, and LDH were detected according to the kit instructions.

#### 2.1.7 Real-Time Quantitative PCR

20 mg of liver tissue was weighed, and then RNA isolater Total RNA Extraction Reagent was added. Perform reverse transcription and amplification according to the instructions of the RT-qPCR reverse transcription and amplification kit. Among them, the mRNAs of *GRP78, PERK, DPR1*, and *LC3* were detected, and the housekeeping gene *GAPDH* was used as a control. Data were analyzed using the 2^−ΔΔCt^ method. The sequences of the gene primers in mice are shown as supplementary material (Supplementary Figure S2).

### 2.2 *In vitro* Study

#### 2.2.1 Cell Culture

The HepaRG cell line, an ideal cell model for hepatotoxicity studies (Wu et al., 2016), was purchased from Beina Chuanglian Biotechnology Company (BNCC340037, Beijing, China), cells were cultured in RPMI 1640 medium containing 10% fetal bovine serum (FBS) and 1% antibiotics, and then cells were cultured in 5% carbon dioxide incubate at 37°C.

#### 2.2.2 Detection of Cell Viability

Cell proliferation was detected by the CCK8 assay. To test the cytotoxicity of TP, HepaRG cells (5 × 10^4^ cells/mL) were placed in 96-well plates for 24 h, then treated with different doses of TP for 12, 24, 36, and 48 h, respectively. After incubation with 10 µl of CCK8 solution for 3 h, the optical density values were determined using a microplate reader (TECAN, Switzerland) at an excitation wavelength of 450 nm.

#### 2.2.3 Detection of Autophagosomes

Acidic autophagic vacuoles were detected by monodansidine cadaverine (MDC) staining using an autophagy detection kit (KeyGEN, Nanjing, China) (Veeran et al., 2017). Cells (1 × 10^5^ cells/mL) were placed in 12-well plates and incubated with different concentrations of TP at 37°C for 24 h. Cells were processed according to the kit instructions and observed and photographed using a fluorescence microscope (Zeiss, Thuringia, Germany) at an excitation wavelength of 355 nm.

### 2.3 Western Blotting

30 mg of mouse liver tissue was weighed or different groups of HepaRG cells were collected, and then 1 ml of lysis buffer was added to disrupt and homogenize, and lysed on ice for 30 min. BCA kit was used for protein concentration detection. 30 µg of protein samples were taken for protein electrophoresis in 10% SDS-PAGE gel. Transfer membrane (300 v, 400 mAh, 1 h) and block (5% BSA), incubate with primary antibody overnight at 4°C. After washing, the secondary antibody was incubated at room temperature for 1 h. After 3 additional washes, exposure was performed using an ECL system (Bio-Rad, California, United States).

### 2.4 Statistics

The data is presented as mean ± standard deviation (*SD*), and statistical analysis was performed using GraphPad Prism 8.0.2. *One-way ANOVA* followed by Dunnett’s post-hoc test and *t-test* were used to compare mean differences between multiple groups or two groups. Differences were considered significant when *p* < 0.05.

## 3 Results

### 3.1 Qingluo Tongbi Formula Alleviated *T. wilfordii*-Induced Hepatotoxicity in C57BL/6J Mice

**FIGURE 1:**
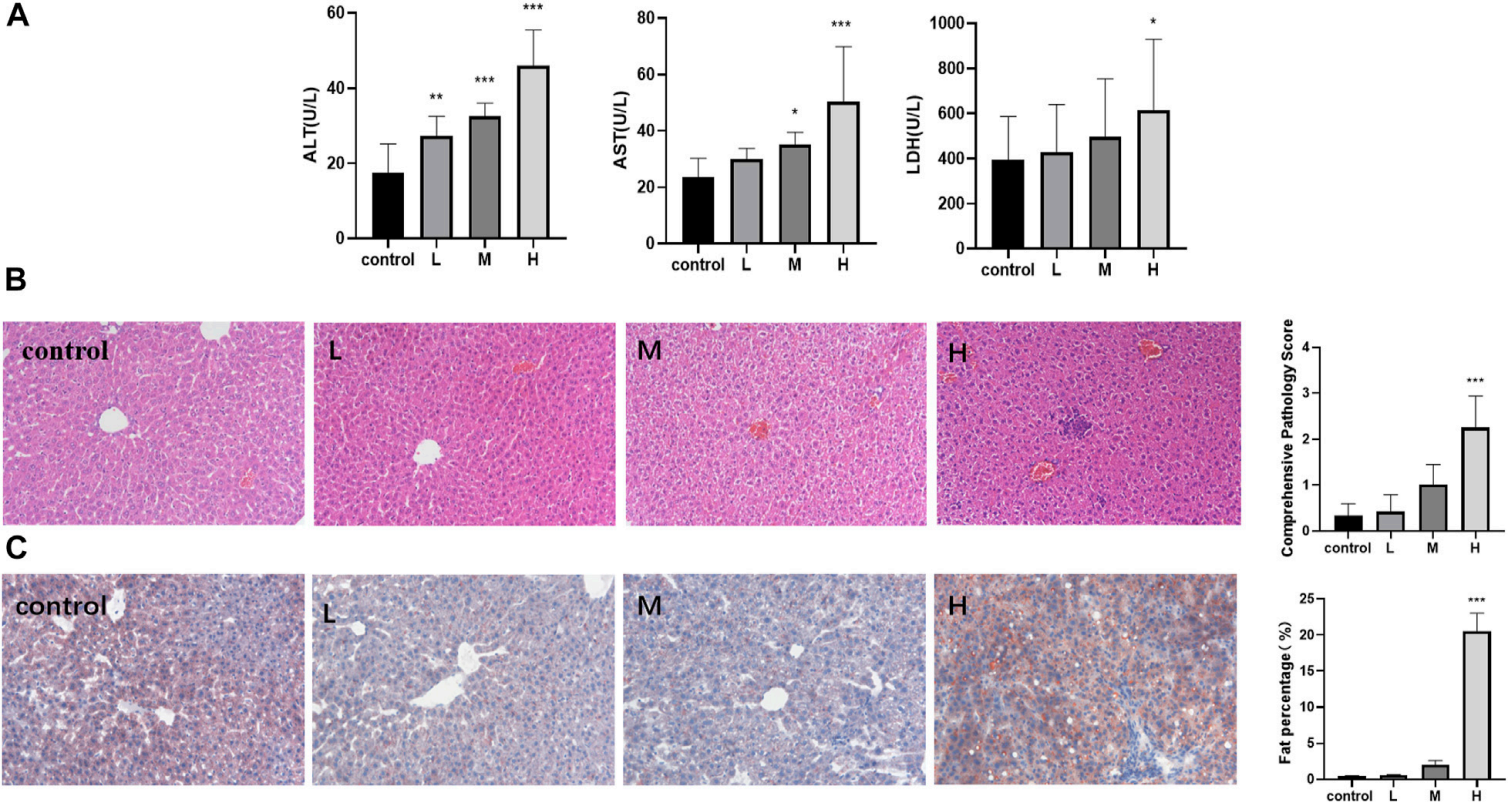
TW induced liver injury in C57BL/6J mice. **(A)** TW-induced elevation of ALT, AST, and LDH. **(B)** TW-induced pathological changes and comprehensive pathological scores by H&E staining. **(C)** TW-induced liver tissue fat accumulation and fat percentage by Oil red O staining. Compared with the control group, ^*^ *p < 0.05,* ^**^ *p < 0.01,* ^***^ *p < 0.001.*

TW increased the levels of ALT, AST, and LDH in a dose-dependent manner in mice, and the three indexes were most significantly elevated in the high-dose TW group (Figure 1A). The pathological results indicated that with the increase of the dose of TW, the liver cells showed different degrees of steatosis, edema degeneration, and inflammatory cell infiltration by H and E staining, and the comprehensive pathological score increased in a dose-dependent manner (Figure 1B). The results by Oil Red O staining indicated that, after administration of TW, the liver cells accumulated fat and the fat percentage of the high-dose TW group was significantly higher than the control (Figure 1C). It can be seen that TW-induced liver damage in mice is dose-dependent. Since the high-dose TW induced the most obvious liver damage, the high dose of TW was used in the subsequent attenuation experiments.

**FIGURE 2:**
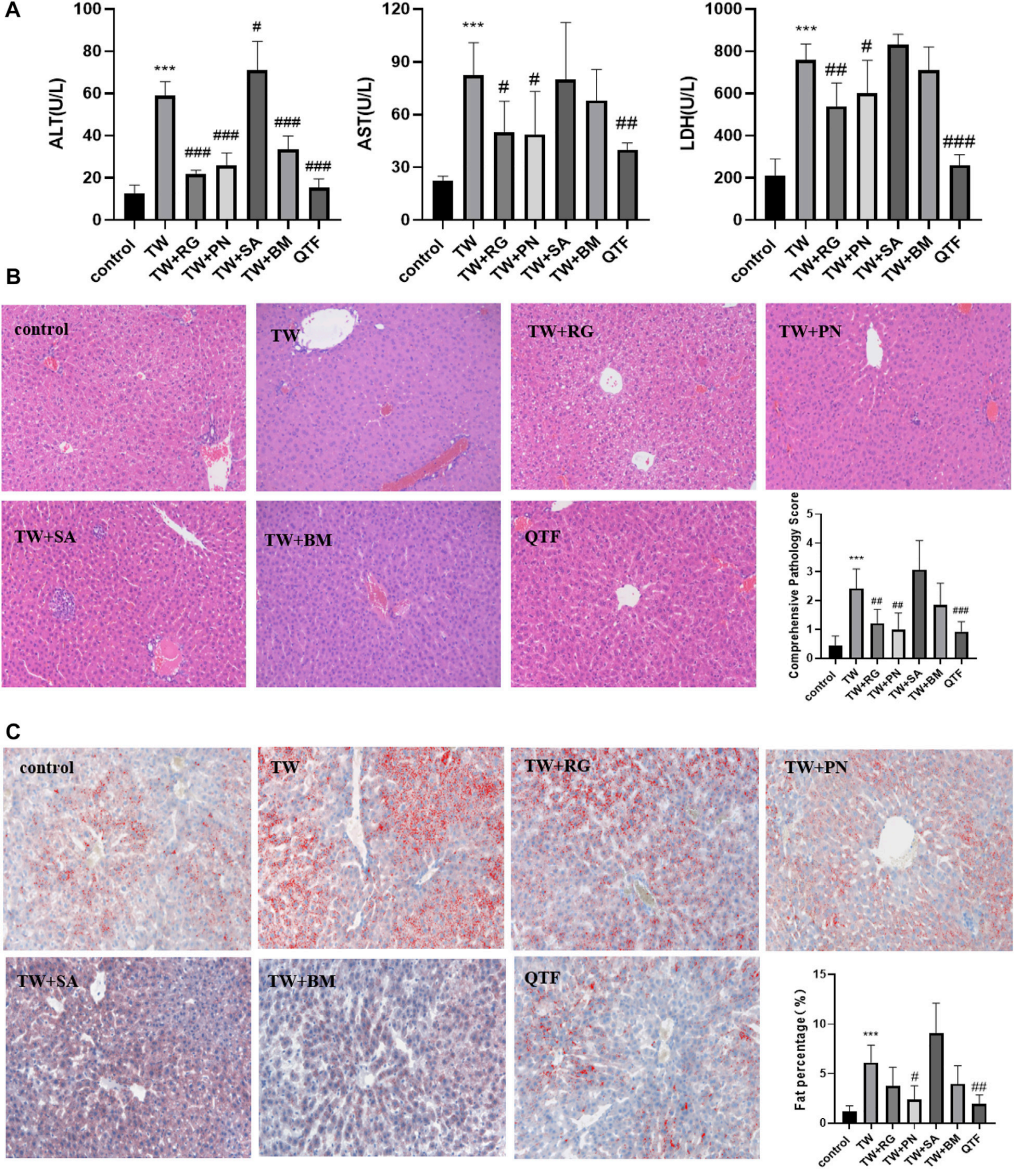
QTF alleviated TW-induced hepatotoxicity in C57BL/6J mice. **(A)** QTF reduced the levels of ALT, AST and LDH caused by TW. **(B)** QTF reduced TW-induced pathological damage and comprehensive pathological score by H&E staining. **(C)** QTF reduced the fat accumulation and fat percentage by Oil red O staining. Compared with the control group, ^*^ *p < 0.05,* ^**^ *p < 0.01,* ^***^ *p < 0.001*; compared with the TW group, ^*#*^ *p < 0.05,* ^*##*^ *p < 0.01,* ^*###*^ *p < 0.001.*

While QTF markedly reduced the elevations of ALT, AST, and LDH caused by TW. Among QTF, RG, and PN significantly reduced the elevation of ALT, AST, and LDH, and BM reduced the TW-induced elevation of ALT; And QTF had the most obvious improvement in the ALT, AST, and LDH caused by TW (Figure 2A). RG, PN, and QTF improved the TW-induced pathological damage of liver cell steatosis, edema degeneration, and inflammatory cell degeneration, and reduced the TW-induced pathological score. And QTF had the most obvious improvement in TW-induced pathological injury and comprehensive pathological score (Figure 2B). Simultaneously, PN and QTF significantly improved the fat accumulation and significantly reduced the fat percentage induced by TW (Figure 2C).

These results showed that QTF reduced the TW-caused hepatotoxicity of mice, and among the drug compatibility of QTF, RG, and PN had the best attenuating effect.

### 3.2 Qingluo Tongbi Formula Attenuated *T. wilfordii*-Induced Excessive Mitophagy and Endoplasmic Reticulum Stress in C57BL/6J Mice

**FIGURE 3:**
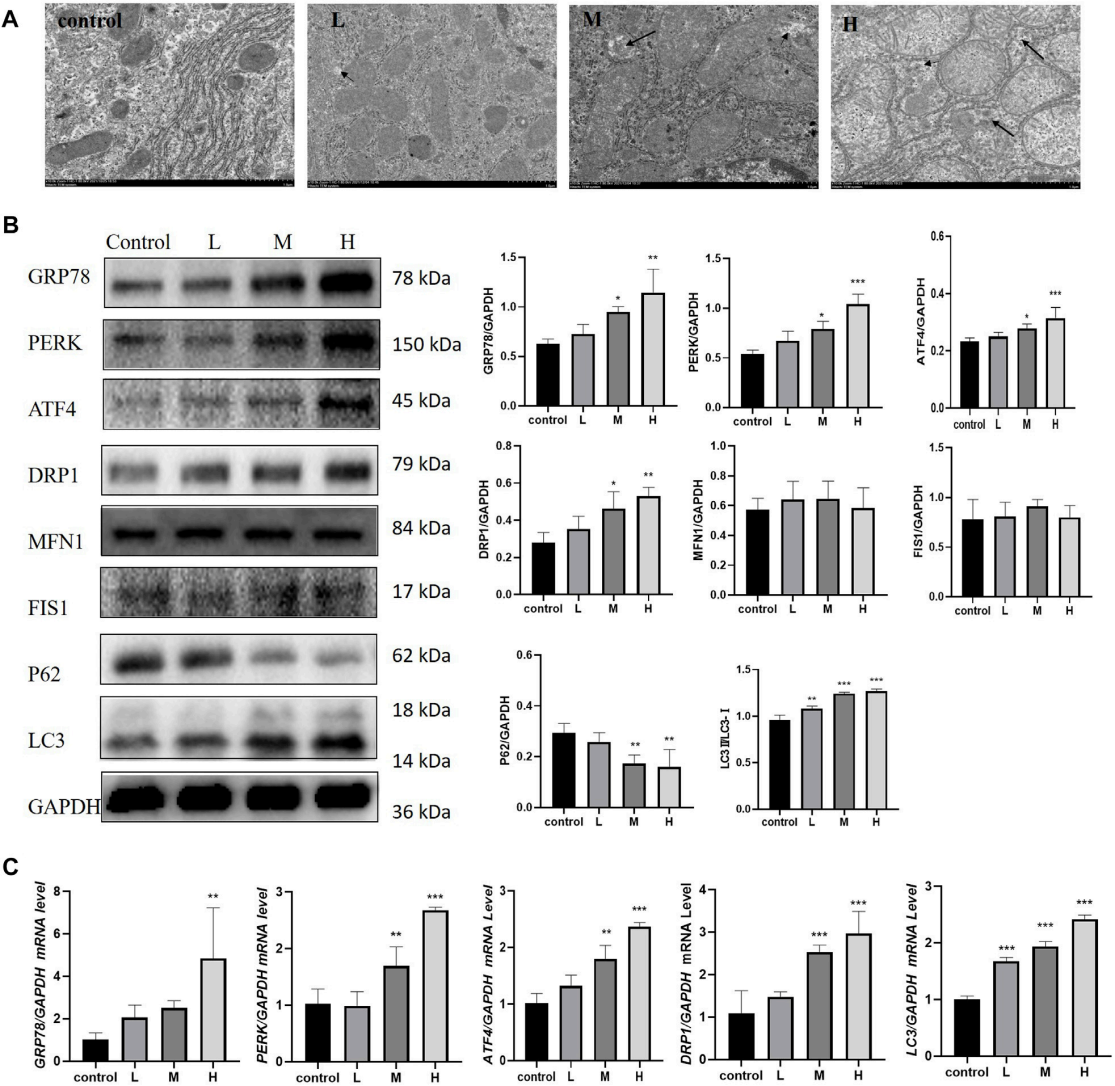
TW induced excessive mitophagy and ERS in C57BL/6J mice. **(A)** TW caused mitochondrial and ER damage, mitochondrial swelling, vacuolization, cristae fragmentation, and increased autophagosomes and autophagolysosomes. The small black arrow represents autophagosomes, and the large black arrow represents autophagolysosomes. 10,000 magnification, Scale bar: 1 µm. **(B)** TW-induced changes in the expression of mitophagy and ERS-related proteins GRP78, PERK, ATF4, DRP1, MFN1, FIS1, P62, and LC3. **(C)** TW-induced changes in mRNA levels of *GRP78, PERK, ATF4, DRP1*, and *LC3*. Compared with the control group, ^*^ *p < 0.05,* ^**^ *p < 0.01,* ^***^ *p < 0.001.*

The results of TEM showed that, TW caused mitochondrial outer membrane and cristae rupture and an increase in autophagosomes and autophagolysosomes in hepatocytes, accompanied by abnormal ER morphology. And the higher the dose of TW was administered, the more severe the damage of mitochondria and ER was shown, and the more autophagosomes and autophagolysosomes were generated (Figure 3A). TW increased the expression levels of mitophagy proteins DRP1 and LC3, and decreased the expression level of P62, while the expression level of MFN1 and FIS1 had no significant changes. It suggested that TW-induced mitophagy was characterized by elevated DRP1. Simultaneously, the expressions of ERS marker protein GRP78 and PERK, and ATF4 were upregulated, indicating that TW induced ERS by activating the PERK-ATF4 pathway in a dose-dependent manner (Figure 3B). And TW simultaneously increased the mRNA levels of *GRP78, PERK, ATF4*, *DRP1*, and *LC3* (Figure 3C). The results indicated that TW induced upregulation of mitophagy and ERS in hepatotoxicity.

**FIGURE 4:**
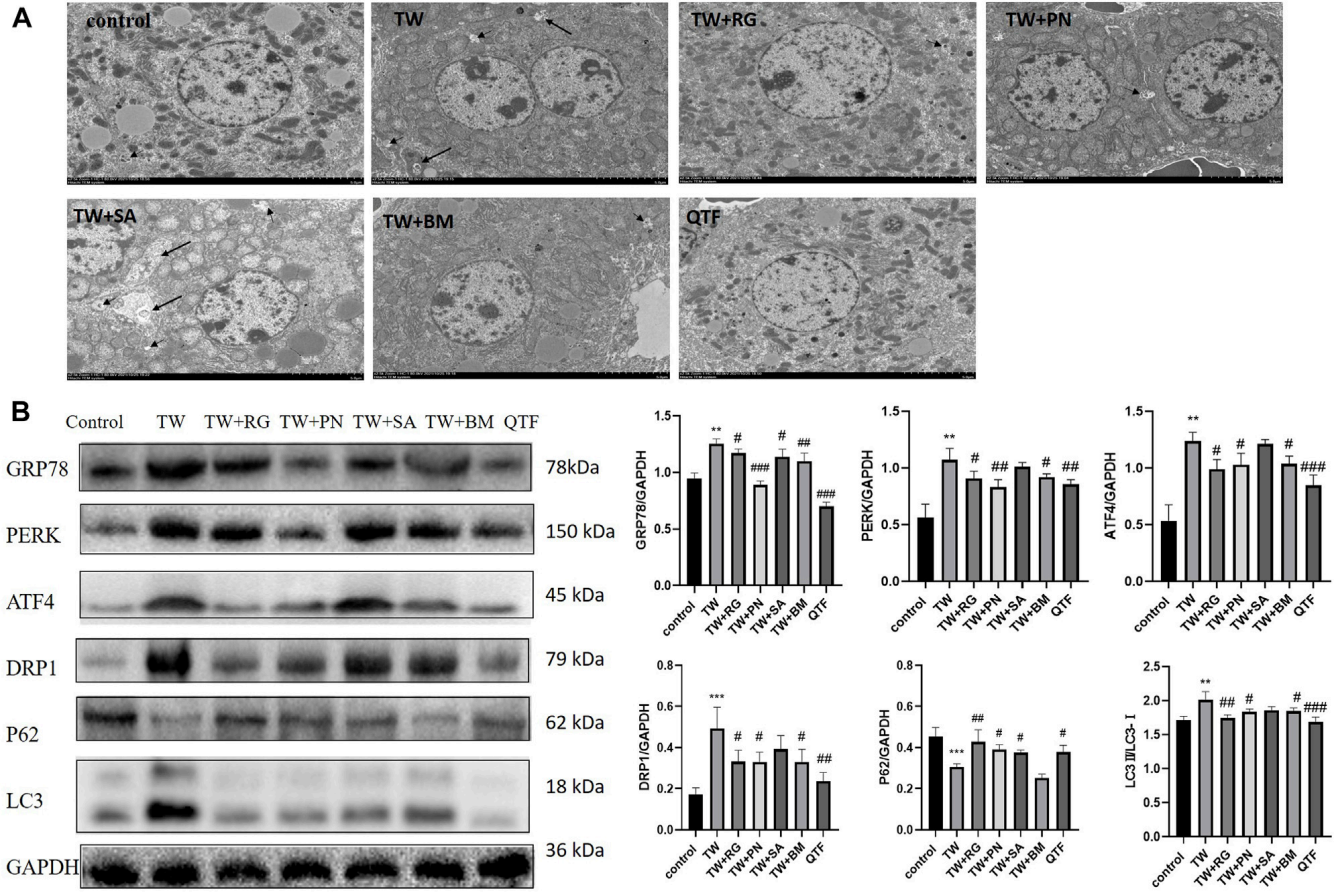
QTF attenuated TW-induced excessive mitophagy and ERS in C57BL/6J mice. **(A)** QTF improved mitochondrial and ER damage induced by TW, and reduced autophagosomes and autophagolysosomes. The small black arrow represents autophagosomes, and the large black arrow represents autophagolysosomes. 2,500 magnification, Scale bar: 5 µm. **(B)** QTF reversed the TW-induced expression level of GRP78, PERK, ATF4, DRP1, P62 and LC3. Compared with the control group, ^*^ *p < 0.05,* ^**^ *p < 0.01,* ^***^ *p < 0.001*; compared with the TW group, ^*#*^ *p < 0.05,* ^*##*^ *p < 0.01,* ^*###*^ *p < 0.001.*

While RG, PN, BM, and QTF improved the TW-induced damage to mitochondria and ER in mice, and reduced autophagosomes and autophagolysosomes (Figure 4A). Simultaneously, RG, PN, BM, and QTF reversed the expression of DRP1, LC3, P62, GRP78, PERK, and ATF4 induced by TW. And among them, QTF improved the most significantly (Figure 4B).

These results indicated that QTF reversed the mitophagy and down-regulated the ERS induced by TW.

### 3.3 Inhibition of Mitophagy and Endoplasmic Reticulum Stress Attenuated Triptolide-Induced Cytotoxicity in HepaRG Cells

In order to further verify the mitophagy and ERS are important mechanisms of TW-induced hepatotoxicity, triptolide (TP), the main active component and also the main toxic component of TW, was used in HepaRG cells.

**FIGURE 5:**
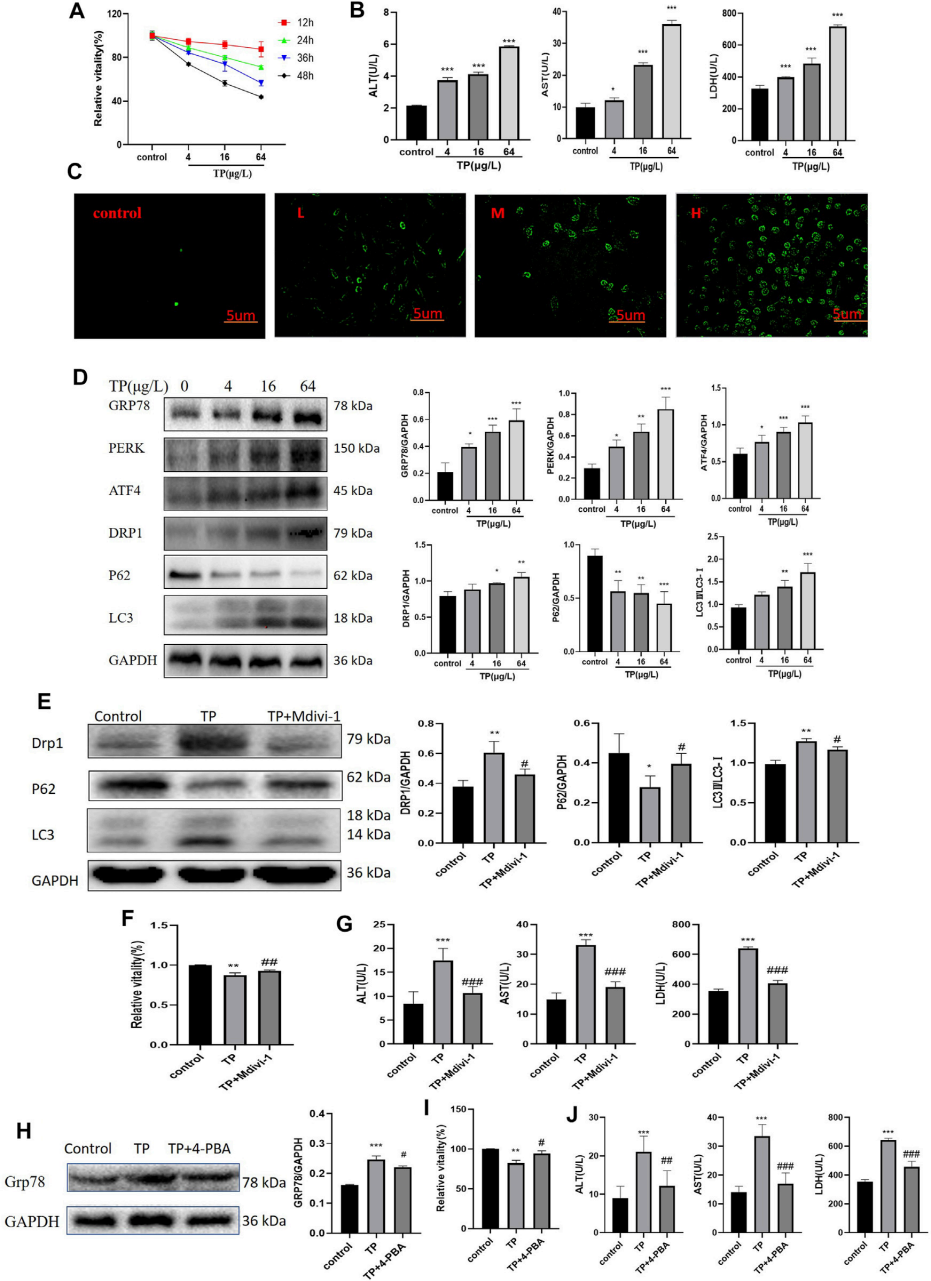
Inhibition of mitophagy and ERS attenuated TP-induced cytotoxicity in HepaRG cells. **(A)** The effect of 4–64 μg/L TP on the viability of HepaRG cells for 12, 24, 36, and 48 h, respectively. **(B)** 4–64 μg/L TP led to the increase of ALT, AST, and LDH indexes in the supernatant of HepaRG cells. **(C)** 4–64 μg/L TP caused the increase of autophagosome production in HepaRG cells. Green fluorescence represented autophagosome. 200 magnification, Scale bar: 5 µm. **(D)** The effect of 4–64 μg/L TP on the expression of GRP78, PERK, ATF4, DRP1, P62, and LC3 in HepaRG cells. **(E)** The effect on the expression levels of DRP1, P62, and LC3 in cells by inhibiting mitophagy by Midivi-1. **(F)** The effect on cell viability by inhibiting mitophagy by Midivi-1. **(G)** The effect on ALT, AST, and LDH indexes in the supernatant of HepaRG cells by inhibiting mitophagy by Midivi-1. **(H)** The effect of 4-PBA on the expression of GRP78 in cells. **(I)** The effect on HepaRG cell viability by inhibiting ERS by 4-PBA. **(J)** The effect on ALT, AST, and LDH indexes in cell supernatant by inhibiting ERS by 4-PBA. Compared with the control group, ^*^ *p < 0.05,* ^**^ *p < 0.01,* ^***^ *p < 0.001*; compared with the TP group, ^*#*^ *p < 0.05,* ^*##*^ *p < 0.01,* ^*###*^ *p < 0.001.*

4–64 μg/L TP resulted in HepaRG cell viability decreased in a time- and dose-dependent manner by CCK8(Figure 5A). And 4–64 μg/L TP led to an increase in ALT, AST, and LDH indexes in the cell supernatant (Figure 5B). Immunofluorescence results showed that the production of autophagosomes increased after TP treatment for 24 h in a dose-dependent manner in HepaRG cells (Figure 5C). Simultaneously, TP led to the up-regulation of DRP1, LC3, GRP78, PERK, and ATF4 (P62 down-regulated) (Figure 5D). The results indicated that TP induced HepaRG cell damage, causing excessive mitophagy and ERS. Since TP at a dose of 16 μg/L induced moderate damage to HepaRG cells, 16 μg/L TP was set for subsequent experiments.

To explore the role of mitophagy in TW-induced hepatotoxicity, we used Mdivi-1 (Deng et al., 2021), a specific inhibitor of mitophagy. When the cells were treated with Mdivi-1, compared to the TP group, the expression levels of DRP1 and LC3 were significantly decreased (that of P62 was significantly increased), suggesting that TP-induced mitophagy was effectively inhibited (Figure 5E). After inhibiting mitophagy, the cell viability was increased (Figure 5F); At the same time, the elevations of ALT, AST, and LDH indexes in the cell supernatant were reduced (Figure 5G).

Likewise, to verify the role of ERS in TW-induced live damage, we applied 4-PBA (Pao et al., 2021), the specific inhibitor of ERS. When HepaRG cells were treated with 4-PBA, the expression level of GRP78 was significantly decreased, suggesting that TP-induced ERS was effectively inhibited (Figure 5H). After inhibiting ERS by 4-PBA, the cell viability induced by TP was increased (Figure 5I); Simultaneously, the elevation of ALT, AST, and LDH indexes in the cell supernatant was decreased (Figure 5J).

These results suggested that excessive mitophagy and ERS were important mechanisms of TW-induced cytotoxicity.

### 3.4 Inhibition of Endoplasmic Reticulum Stress Attenuated TP-Induced Excessive Mitophagy in HepaRG Cells

**FIGURE 6:**
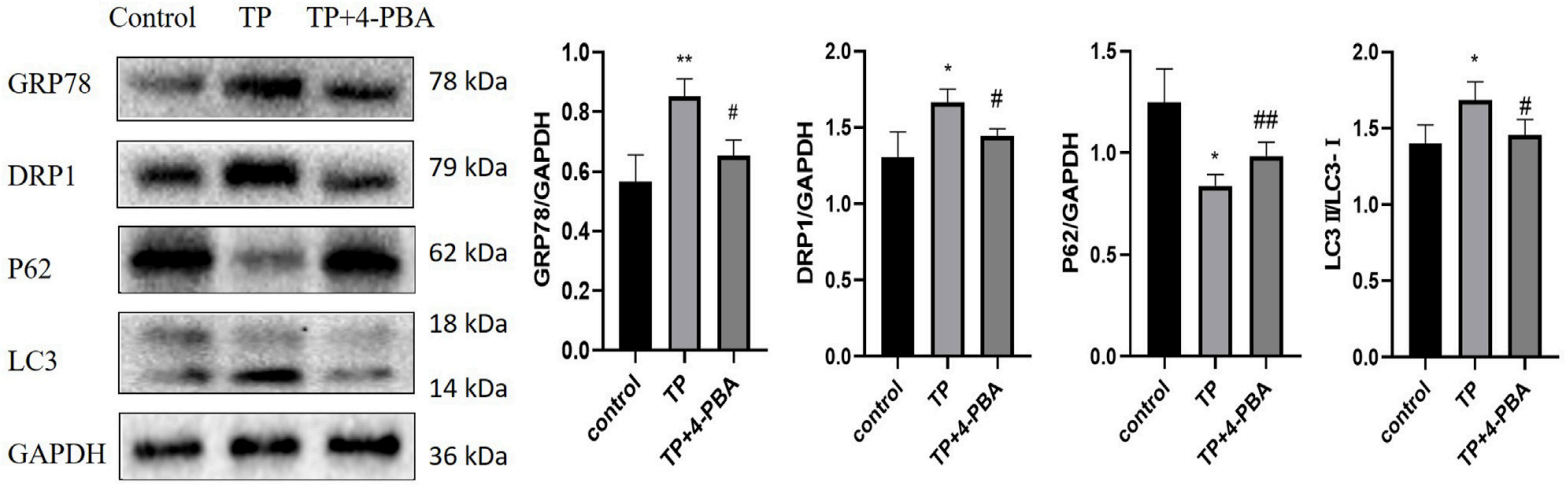
Inhibition of ERS attenuated TP-induced excessive mitophagy in HepaRG cells. The effect on the expression levels of DRP1, P62, and LC3 by inhibiting ERS by 4-PBA. Compared with the control group, ^*^ *p < 0.05,* ^***^ *p < 0.001*; compared with the TP group, ^*#*^ *p < 0.05,* ^*##*^ *p < 0.01.*

To further explore the relationship between ERS and mitophagy in TW-induced hepatotoxicity, 4-PBA was used. When ERS was inhibited by 4-PBA in HepaRG cells, the expression level of GRP78 was down-regulated, and the expression levels of DRP1 and LC3 were also decreased (that of P62 was increased), indicating that ERS regulated mitophagy (Figure 6).

### 3.5 Inhibition of the PERK-ATF4 Pathway Downregulated TP-Induced Excessive Mitophagy in HepaRG Cells

**FIGURE 7:**
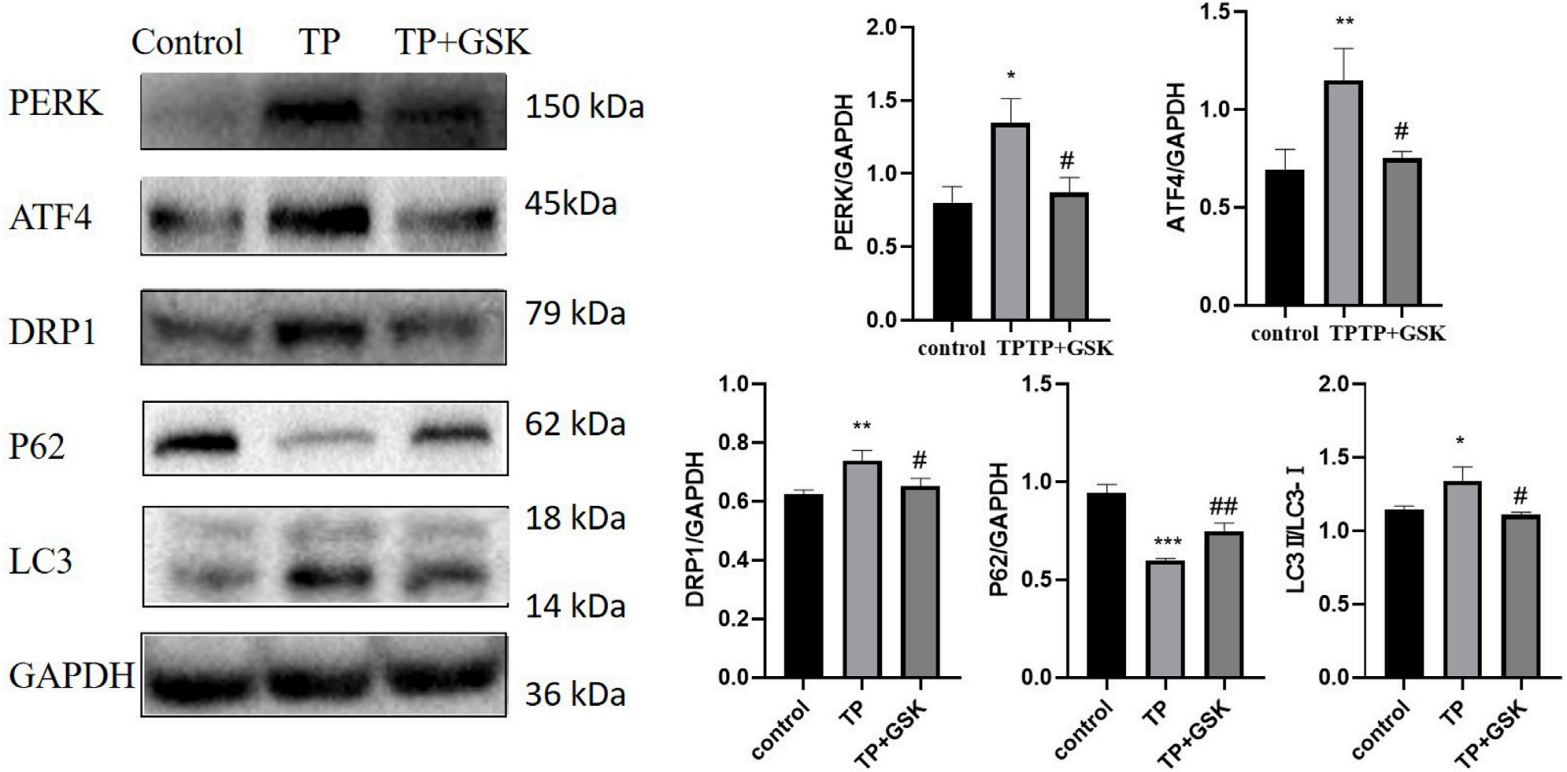
Inhibition of the PERK-ATF4 pathway downregulated TP-induced excessive mitophagy in HepaRG cells. Effects on the expression of ATF4, DRP1, P62, and LC3 by inhibiting PERK by GSK2656157. Compared with the control group, ^*^ *p < 0.05,* ^**^ *p < 0.01,* ^***^ *p < 0.001*; compared with the TP group, ^*#*^ *p < 0.05,* ^*##*^ *p < 0.01.*

To further verify the relationship between the PERK-ATF4 pathway and mitophagy, we applied GSK2656157 (Axten et al., 2013), the specific inhibitor of PERK. When the expression level of PERK was down-regulated by GSK2656157, the expression of ATF4, a key protein downstream of PERK, was down-regulated, and simultaneously, the expression levels of DRP1 and LC3 were decreased (that of P62 was increased) (Figure 7), indicating that the PERK-ATF4 pathway regulates mitophagy in TP-induced cell damage.

### 3.6 The Pharmacodynamics Combination of Qingluo Tongbi Formula Reduced TP-Induced Damage by DownRegulating Excessive Mitophagy Through the PERK-ATF4 Pathway in HepaRG Cells

To further verify that QTF reduces TW-caused hepatotoxicity by inhibiting ERS by the PERK-ATF4 pathway and then downregulating excessive mitophagy, we used TP, catalpol (CAT, the main active ingredient of RG), and panax notoginseng saponins (PNS, the main active ingredient of PN), which are the main active ingredients of QTF.

**FIGURE 8:**
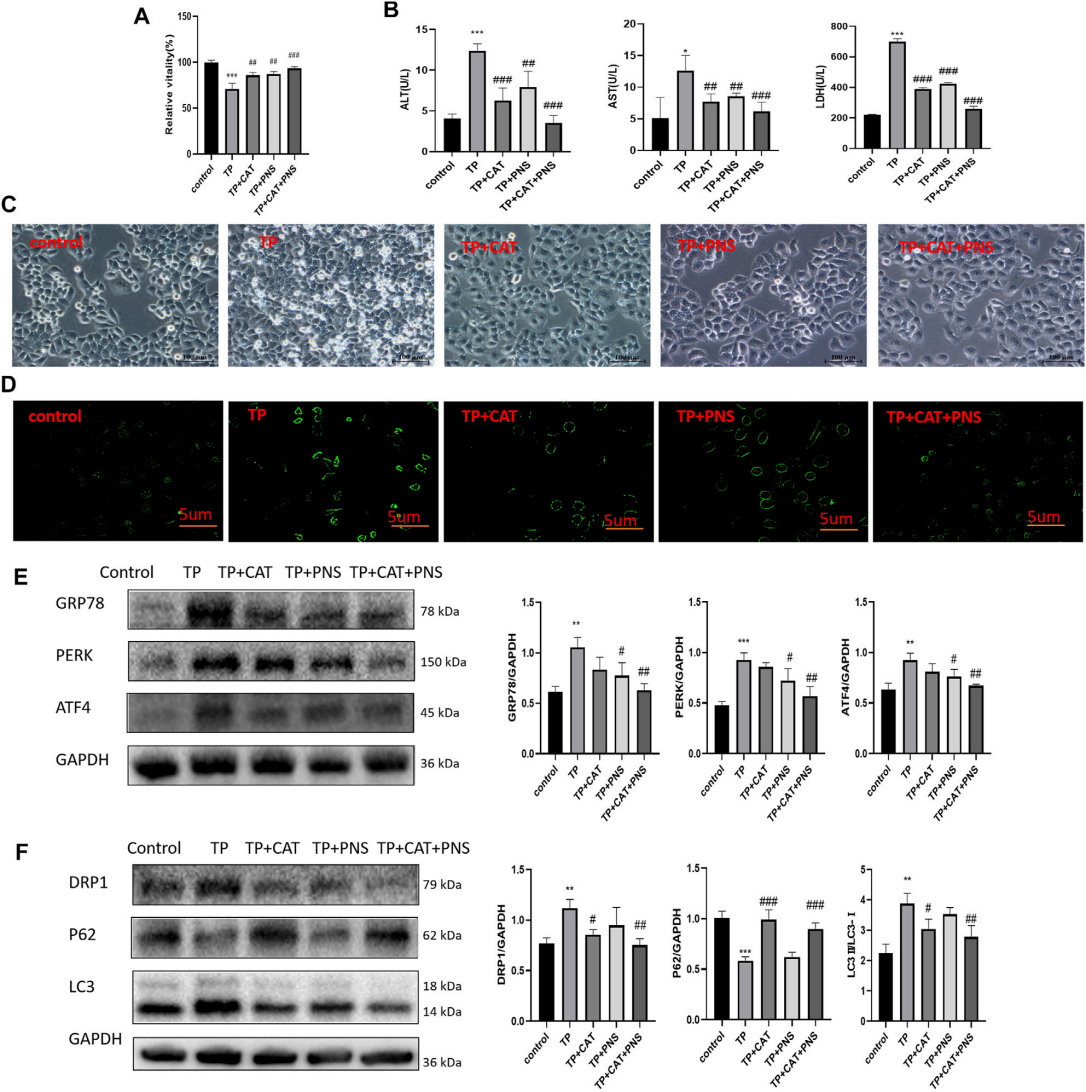
The pharmacodynamics combination of QTF reduced TP-induced damage by down-regulating excessive mitophagy by the PERK-ATF4 pathway in HepaRG cells. **(A)** CAT and PNS improved the TP-induced reduction of HepaRG cell viability. **(B)** CAT and PNS improved the levels of ALT, AST, and LDH in the supernatant of HepaRG cells induced by TP. **(C)** CAT and PNS improved the TP-induced morphological damage to HepaRG cells. 200 magnification, Scale bar: 10 µm. **(D)** CAT and PNS reduced the TP-induced increase of autophagosomes in HepaRG cells. The green fluorescence represents autophagosomes. 200 magnification, Scale bar: 5 µm. **(E)** CAT and PNS reduced the expression level of GRP78, PERK, and ATF4 in HepaRG cells induced by TP. **(F)** CAT and PNS reversed the expression level of DRP1, P62, and LC3 in HepaRG cells induced by TP. Compared with the control group, ^*^ *p < 0.05,* ^**^ *p < 0.01,* ^***^ *p < 0.001*; compared with the TP group, ^*#*^ *p < 0.05,* ^*##*^ *p < 0.01.*

Firstly, the safe dosage of CAT and PNS was screened in HepaRG cells by CCK8, respectively. Then we screened the respective optimal protective doses of CAT and PNS, and found that when CAT was 80 μg/l and PNS was 10 μg/l, their respective protective effect was the most obvious (Supplementary Figure S3). The combination of CAT and PNS improved the viability of HepaRG cells induced by TP (Figure 8A); Simultaneously, they significantly reduced the ALT, AST, and LDH in the supernatant of HepaRG cells induced by TP (Figure 8B). CAT and PNS also significantly ameliorated TP-induced cell morphological damage and death (Figure 8C); Meanwhile, they also reduced TP-induced autophagosomes in HepaRG cells (Figure 8D). PNS was superior to CAT in inhibiting the expression levels of GRP78, PERK, and ATF4 (Figure 8E), while CAT was superior to PNS in reversing the expression levels of DRP1, P62, and LC3 (Figure 8F). The combination of CAT and PNS had the most obvious regulatory effect on the PERK-ATF4 pathway and mitophagy.

## 4 Discussion

Mitochondria is the “energy reservoir” of cells, providing the necessary energy for various cellular activities through oxidative phosphorylation (Annesley & Fisher 2019). Studies have shown that mitochondrial damage and dysfunction play a causative role in drug-induced liver injury (Fromenty & Pessayre 1995; Pessayre et al., 1999; Lin et al., 2019). Mitophagy, as an important type of selective autophagy, is the main way to remove damaged mitochondria and determines the normal performance of mitochondrial quantity and function (Ashrafi & Schwarz 2013). However, excessive mitophagy induced by drugs and other stimuli results in abnormal mitochondrial number and energy metabolism, causing apoptosis or autophagic death. Mitochondria is an organelle that constantly undergoes division and fusion. The balance between mitochondrial division and fusion maintains its normal shape and function and is an important basis for ensuring the normal progress of various physiological activities of cells (Yoo & Jung 2018). Mitochondrial fragmentation resulting from an imbalance of mitochondrial fission and fusion is a prerequisite for mitophagy (Chen et al., 2016). Some research has shown that, mitochondrial fission-related factors and degradation of mitochondrial fusion-related factors are required for mitophagy (Shirihai et al., 2015). In mammals, the main proteins that regulate mitochondrial fission are dynamin-related protein 1 (DRP1), mitochondrial fission 1 (FIS1), etc., The proteins that regulate mitochondrial fusion mainly include mitochondrial fusion (MFN, which has two isoforms, MFN1 and MFN2), optic atrophy 1 (OPA1) (Chan 2006). DRP1 is mainly located in the cytoplasm and is a member of the protein dynein superfamily, also known as dynein 1 (Dynamin 1, DNM1L). Some studies suggest that DRP1 is required for mitochondrial fission (Fonseca et al., 2019) and is also the key marker of mitophagy in TP-induced hepatotoxicity (Hasnat et al., 2019). Our study proved that in mice treated with TW, the expression of mitophagy-related proteins was abnormal, which was characterized by the increased expression level of DRP1, while the expression levels of MFN1 and FIS1 did not change significantly. QTF could significantly reverse TW-induced expression levels of mitophagy proteins DRP1, P62, and LC3-Ⅱ. And QTF could intuitively improve TW-induced mitochondrial morphological damage and reduce autophagosomes and autophagolysosomes by TEM. To further study the important role of mitophagy in the TW-induced hepatotoxicity, we used, TP, CAT, and PNS to verify the mechanism with the mitophagy inhibitor Midivi-1 in HepaRG cells. The results showed that, when mitophagy was inhibited by Midivi-1, the expression levels of DRP1, LC3-Ⅱ were down-regulated (that of P62 was up-regulated), and cell viability and liver function indicators ALT, AST, and LDH were improved, suggesting that excessive mitophagy was an important mechanism of TW-induced hepatotoxicity.

Furthermore, TW-caused hepatotoxicity was also accompanied by morphological damage of the ER and the protein and gene levels of Glucose-regulated protein 78 (GRP78) and protein kinase R-like endoplasmic reticulum kinase (PERK) increased, while QTF significantly improved the TW-induced morphological damage of ER and reduced the expression level of GRP78 and PERK. When physical, chemical, and other factors stimulate the body, too many unfolded/misfolded proteins accumulate in the ER, causing ERS and triggering unfolded protein response (UPR) (Ron & Walter 2007); If UPR fails to maintain balance, the cell survival mechanism will turn to the death mechanism (Hetz 2012; Kraskiewicz & FitzGerald 2012). When cells initiate the UPR, GRP78 expression is upregulated, depolymerized and activated to further correct the protein folding of erroneous proteins while activating mechanisms such as autophagy and apoptosis (Lu et al., 2020). In *in vitro* experiments, we used 4-PBA for mechanistic studies to further clarify the role of ERS in TW-induced hepatotoxicity. When ERS was inhibited by 4-PBA, the cell viability and the levels of ALT, AST, and LDH improved, indicating that ERS was also an important mechanism of TW-induced hepatotoxicity, while QTF inhibited the TW-caused ERS.

A cell is an organic whole, like the human body. As the largest membranous organelle in cells, the ER is an important site for protein synthesis, processing and modification (Wang & Kaufman 2016). It can coordinately regulate physiological activities such as autophagy with mitochondria through MAMs. However, it is not clear that how ERS affects mitophagy and whether QTF reduces the hepatotoxicity of TW is related to ERS and mitophagy. We inhibited ERS by 4-PBA to investigate the link between ERS and mitophagy in TW-induced hepatotoxicity; When ERS was inhibited by 4-PBA, the expression levels of DRP1 and LC3-II were downregulated (that of P62 was upregulated), demonstrating that ERS regulates mitophagy. It is known that under ERS, cells mainly initiate UPR through three pathways: PERK, inositol-requiring enzyme 1 (IRE1), and activated transcription factor 6 (ATF6). The PERK pathway is the preferred activation pathway induced by ERS (Fung et al., 2015). PERK is a transmembrane protein located in the ER. When ERS occurs, PERK dissociates from GRP78 and is activated by autophosphorylation, and further activates Activating Transcription Factor 4 (ATF4) by phosphorylating eukaryotic initiation factor 2α (eIF2α) (Krishnamoorthy et al., 2014). ATF4, located downstream of the PERK pathway, can regulate the expression of multiple autophagy-related genes, including *LC3B, ATG5, ATG7*, and *Beclin1*, and plays an extremely important role in regulating autophagy (Rzymski et al., 2009). Simultaneously, under persistent ERS, ATF4 also induces apoptosis by degrading XIAP and cooperating with C/EBP-homologous protein (CHOP) (B'Chir et al., 2013). The results of our study demonstrated that the PERK-ATF4 pathway was activated in TW-induced hepatotoxicity. To further verify that ERS regulates mitophagy by the PERK-ATF4 pathway, we applied GSK2656157. When the PERK-ATF4 pathway was inhibited by GSK2656157, the expression levels of DRP1 and LC3-Ⅱ decreased (that of P62 increased), suggesting the PERK-ATF4 pathway was an important pathway for ERS to regulate mitophagy.

The detoxification effects of RG and PN in QTF were the most obvious. *In vitro* studies showed that CAT, the main active ingredient of RG, significantly inhibited the expression of mitophagy proteins DRP1 and LC3-II induced by TP (down-regulated the expression of P62), while PNS, the main active ingredient of PN, significantly inhibited the expression of GRP78, PERK and ATF4 induced by TP. CAT is superior to PNS in regulating mitophagy, and PNS is superior to CAT in regulating ERS.

However, how does DRP1 regulate mitophagy in TW-induced hepatotoxicity? And whether QTF alleviates TW-induced hepatotoxicity by regulating mitophagy by the PINK1-Parkin pathway? These are not quite clear yet and our team will conduct further research in the future.

## 5 Conclusion

Our study shows that, TW-induced hepatotoxicity is associated with excessive mitophagy and ERS, and ERS regulates mitophagy by the PERK-ATF4 pathway; QTF downregulates excessive mitophagy to reduce the TW-induced hepatotoxicity by inhibiting ERS by the PERK-ATF4 pathway. QTF differentially regulates different sites of the PERK-ATF4 pathway and mitophagy through different components to exert the attenuation effect.

## Data Availability

The raw data supporting the conclusion of this article will be made available by the authors, without undue reservation.

## Abbreviations

ALT, Alanine aminotransferase; AST, Aspartate aminotransferase; ATF4, Activating transcription factor 4; BM, *Bombyx mori L;* CAT, Catalpol; CCK8, Cell Counting Kit-8; DRP1, Dynamin-related protein 1; ERS, Endoplasmic reticulum; FIS1, Fission 1; GRP78, Glucose-regulated protein 78; H and E, Hematoxylin-Eosin staining; LC3, Microtubule-associated protein 1A/1B-light chain 3; LDH, Lactate dehydrogenase; Mdivi-1, Mitochondrial division inhibitor 1; MFN1, Mitofusin-1; PERK, PKR-like endoplasmic reticulum kinase; PN, *Panax notoginseng* (Burkill) F.H.Chen; PNS, Panax notoginseng saponins; QTF, Qingluotongbi formula; RA, Rheumatoid arthritis; RG, *Rehmannia* glutinosa (Gaertn.) DC; TW, *Tripterygium wilfordii* Hook. F; TCM, traditional Chinese medicine; TP, Triptolide; UPR, Unfolded protein response; 4-PBA, 4-Phenylbutyric acid;

## References

[B1] Annesley S. J. Fisher P. R. (2019). Mitochondria in Health and Disease. Cells 8, 680. 10.3390/cells8070680 PMC6678092 31284394

[B2] Ashrafi G. Schwarz T. L. (2013). The Pathways of Mitophagy for Quality Control and Clearance of Mitochondria. Cell. Death Differ. 20, 31–42. 10.1038/cdd.2012.81 22743996 PMC3524633

[B3] Axten J. M. Romeril S. P. Shu A. Ralph J. Medina J. R. Feng Y. (2013). Discovery of GSK2656157: An Optimized PERK Inhibitor Selected for Preclinical Development. ACS Med. Chem. Lett. 4, 964–968. 10.1021/ml400228e 24900593 PMC4027568

[B4] B'Chir W. Maurin A. C. Carraro V. Averous J. Jousse C. Muranishi Y. (2013). The eIF2α/ATF4 Pathway Is Essential for Stress-Induced Autophagy Gene Expression. Nucleic Acids Res. 41, 7683–7699. 10.1093/nar/gkt563 23804767 PMC3763548

[B5] Brunt E. M. (2000). Grading and Staging the Histopathological Lesions of Chronic Hepatitis: the Knodell Histology Activity Index and beyond. Hepatology 31, 241–246. 10.1002/hep.510310136 10613753

[B6] Chan D. C. (2006). Mitochondria: Dynamic Organelles in Disease, Aging, and Development. Cell. 125, 1241–1252. 10.1016/j.cell.2006.06.010 16814712

[B7] Chen M. Chen Z. Wang Y. Tan Z. Zhu C. Li Y. (2016). Mitophagy Receptor FUNDC1 Regulates Mitochondrial Dynamics and Mitophagy. Autophagy 12, 689–702. 10.1080/15548627.2016.1151580 27050458 PMC4836026

[B8] Deng Y. Li S. Chen Z. Wang W. Geng B. Cai J. (2021). Mdivi-1, a Mitochondrial Fission Inhibitor, Reduces Angiotensin-II- Induced Hypertension by Mediating VSMC Phenotypic Switch. Biomed. Pharmacother. 140, 111689. 10.1016/j.biopha.2021.111689 34004510

[B9] Fonseca T. B. Sánchez-Guerrero Á. Milosevic I. Raimundo N. (2019). Mitochondrial Fission Requires DRP1 but Not Dynamins. Nature 570, E34–e42. 10.1038/s41586-019-1296-y 31217603

[B10] Fromenty B. Pessayre D. (1995). Inhibition of Mitochondrial Beta-Oxidation as a Mechanism of Hepatotoxicity. Pharmacol. Ther. 67, 101–154. 10.1016/0163-7258(95)00012-6 7494860

[B11] Fung T. S. Torres J. Liu D. X. (2015). The Emerging Roles of Viroporins in ER Stress Response and Autophagy Induction during Virus Infection. Viruses 7, 2834–2857. 10.3390/v7062749 26053926 PMC4488716

[B12] Hasnat M. Yuan Z. Naveed M. Khan A. Raza F. Xu D. (2019). Drp1-associated Mitochondrial Dysfunction and Mitochondrial Autophagy: a Novel Mechanism in Triptolide-Induced Hepatotoxicity. Cell. Biol. Toxicol. 35, 267–280. 10.1007/s10565-018-9447-8 30542779

[B13] Hetz C. (2012). The Unfolded Protein Response: Controlling Cell Fate Decisions under ER Stress and beyond. Nat. Rev. Mol. Cell. Biol. 13, 89–102. 10.1038/nrm3270 22251901

[B14] Kraskiewicz H. FitzGerald U. (2012). InterfERing with Endoplasmic Reticulum Stress. Trends Pharmacol. Sci. 33, 53–63. 10.1016/j.tips.2011.10.002 22112465

[B15] Krishnamoorthy J. Rajesh K. Mirzajani F. Kesoglidou P. Papadakis A. I. Koromilas A. E. (2014). Evidence for eIF2α Phosphorylation-independent Effects of GSK2656157, a Novel Catalytic Inhibitor of PERK with Clinical Implications. Cell. Cycle 13, 801–806. 10.4161/cc.27726 24401334 PMC3979916

[B16] Lin L. Liu Y. Fu S. Qu C. Li H. Ni J. (2019). Inhibition of Mitochondrial Complex Function-The Hepatotoxicity Mechanism of Emodin Based on Quantitative Proteomic Analyses. Cells 8, 263. 10.3390/cells8030263 PMC6468815 30897821

[B17] Liu T. Y. Zhou L. L. Zhou C. Liu Z. P. Chen C. Feng Z. (2015). Inhibition Mechanism of Qingluo Tongbi Granule () on Osteoclast Differentiation Induced by Synovial Fibroblast and Monocytes Co-culture in Adjuvant-Induced Arthritic Rats. Chin. J. Integr. Med. 21, 291–298. 10.1007/s11655-014-1839-x 25182154

[B18] Lu G. Luo H. Zhu X. (2020). Targeting the GRP78 Pathway for Cancer Therapy. Front. Med. (Lausanne) 7, 351. 10.3389/fmed.2020.00351 32850882 PMC7409388

[B19] Lv H. Jiang L. Zhu M. Li Y. Luo M. Jiang P. (2019). The Genus Tripterygium: A Phytochemistry and Pharmacological Review. Fitoterapia 137, 104190. 10.1016/j.fitote.2019.104190 31163199

[B20] Pao H. P. Liao W. I. Tang S. E. Wu S. Y. Huang K. L. Chu S. J. (2021). Suppression of Endoplasmic Reticulum Stress by 4-PBA Protects Against Hyperoxia-Induced Acute Lung Injury via Up-Regulating Claudin-4 Expression. Front. Immunol. 12, 674316. 10.3389/fimmu.2021.674316 34122432 PMC8194262

[B21] Pessayre D. Mansouri A. Haouzi D. Fromenty B. (1999). Hepatotoxicity Due to Mitochondrial Dysfunction. Cell. Biol. Toxicol. 15, 367–373. 10.1023/a:1007649815992 10811531

[B22] Ramos M. I. Karpus O. N. Broekstra P. Aarrass S. Jacobsen S. E. Tak P. P. (2015). Absence of Fms-like Tyrosine Kinase 3 Ligand (Flt3L) Signalling Protects against Collagen-Induced Arthritis. Ann. Rheum. Dis. 74, 211–219. 10.1136/annrheumdis-2013-203371 24064002

[B23] Ron D. Walter P. (2007). Signal Integration in the Endoplasmic Reticulum Unfolded Protein Response. Nat. Rev. Mol. Cell. Biol. 8, 519–529. 10.1038/nrm2199 17565364

[B24] Rzymski T. Milani M. Singleton D. C. Harris A. L. (2009). Role of ATF4 in Regulation of Autophagy and Resistance to Drugs and Hypoxia. Cell. Cycle 8, 3838–3847. 10.4161/cc.8.23.10086 19887912

[B25] Shirihai O. S. Song M. Dorn G. W. 2nd (2015). How Mitochondrial Dynamism Orchestrates Mitophagy. Circ. Res. 116, 1835–1849. 10.1161/circresaha.116.306374 25999423 PMC4443843

[B26] Veeran S. Shu B. Cui G. Fu S. Zhong G. (2017). Curcumin Induces Autophagic Cell Death in Spodoptera Frugiperda Cells. Pestic. Biochem. Physiol. 139, 79–86. 10.1016/j.pestbp.2017.05.004 28595926

[B27] Wang M. Kaufman R. J. (2016). Protein Misfolding in the Endoplasmic Reticulum as a Conduit to Human Disease. Nature 529, 326–335. 10.1038/nature17041 26791723

[B28] Wu Y. Geng X. C. Wang J. F. Miao Y. F. Lu Y. L. Li B. (2016). The HepaRG Cell Line, a Superior *In Vitro* Model to L-02, HepG2 and hiHeps Cell Lines for Assessing Drug-Induced Liver Injury. Cell. Biol. Toxicol. 32, 37–59. 10.1007/s10565-016-9316-2 27027780

[B29] Yang P. Qian F. Zhang M. Xu A. L. Wang X. Jiang B. (2020). Zishen Tongluo Formula Ameliorates Collagen-Induced Arthritis in Mice by Modulation of Th17/Treg Balance. J. Ethnopharmacol. 250, 112428. 10.1016/j.jep.2019.112428 31783137

[B30] Yoo S. M. Jung Y. K. (2018). A Molecular Approach to Mitophagy and Mitochondrial Dynamics. Mol. Cells 41, 18–26. 10.14348/molcells.2018.2277 29370689 PMC5792708

[B31] Yu Z. Feng Z. Fu L. Wang J. Li C. Zhu H. (2022). Qingluotongbi Formula Regulates the LXRα-ERS-SREBP-1c Pathway in Hepatocytes to Alleviate the Liver Injury Caused by Tripterygium Wilfordii Hook. F. J. Ethnopharmacol. 287, 114952. 10.1016/j.jep.2021.114952 34968661

[B32] Zhang L. Li C. Fu L. Yu Z. Xu G. Zhou J. (2022). Protection of Catalpol against Triptolide-Induced Hepatotoxicity by Inhibiting Excessive Autophagy via the PERK-ATF4-CHOP Pathway. PeerJ 10, e12759. 10.7717/peerj.12759 35036109 PMC8742543

[B33] Zhang Q. Li Y. Liu M. Duan J. Zhou X. Zhu H. (2018). Compatibility with Panax Notoginseng and Rehmannia Glutinosa Alleviates the Hepatotoxicity and Nephrotoxicity of Tripterygium Wilfordii via Modulating the Pharmacokinetics of Triptolide. Int. J. Mol. Sci. 19. 10.3390/ijms19010305 PMC5796250 29351251

