# Supplementary material for: Qingluo Tongbi Formula Alleviates Hepatotoxicity Induced by Tripterygium wilfordii Hook. F. by Regulating Excessive Mitophagy Through the PERK-ATF4 Pathway
Source: Front Pharmacol. 2022 Jul 7;13:918466. doi: 10.3389/fphar.2022.918466 (PMC9301126; doi:10.3389/fphar.2022.918466)
Supplement: Supplementary file 1 [file DataSheet1.doc]

**
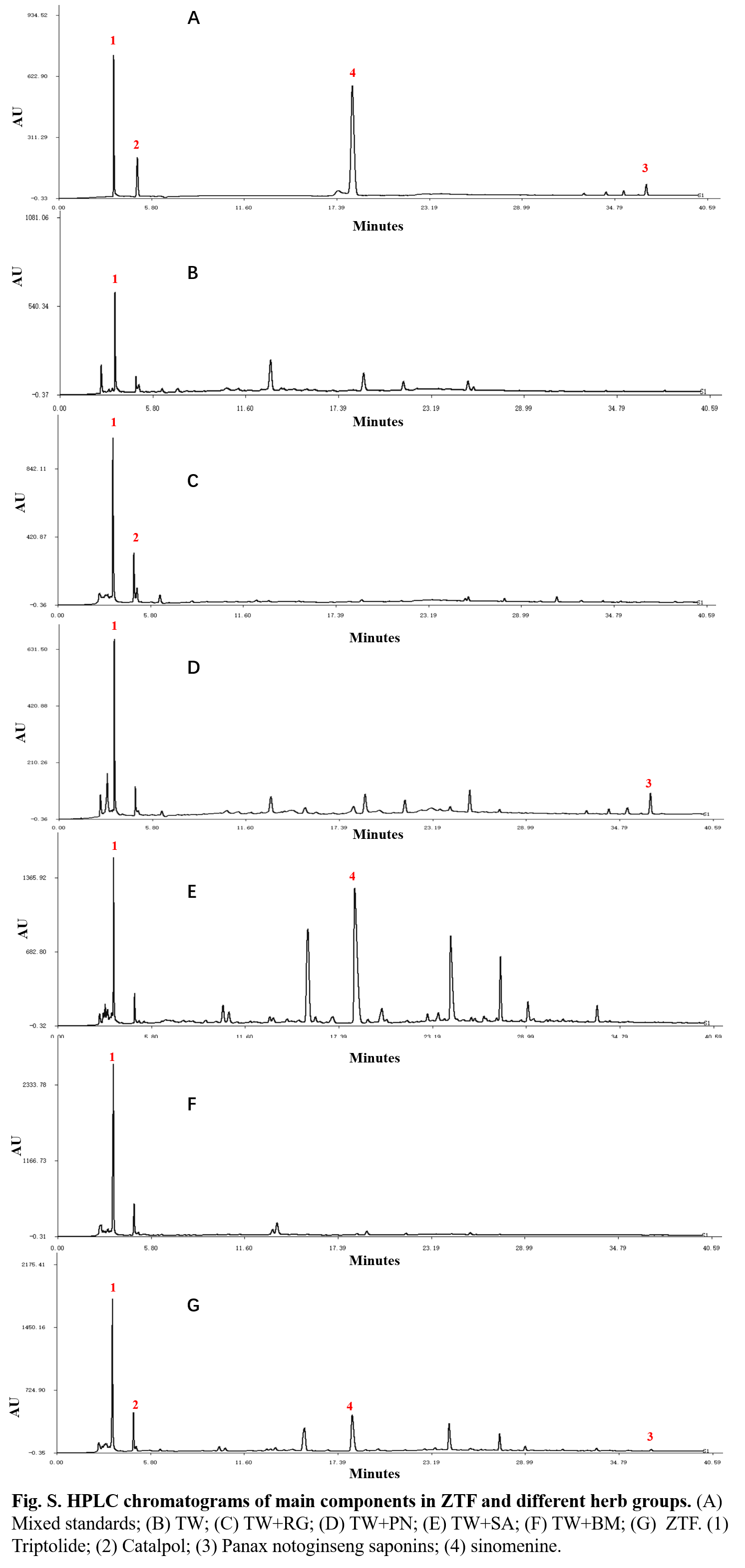
**

**Figure S1. HPLC chromatograms of the main components in ZTF and different herb groups.**

(A) Mixed standards; (B) TW; (C) TW+RG; (D) TW+PN; (E) TW+SA; (F) TW+BM; (G) ZTF. (1) Triptolide; (2) Catalpol; (3) Panax notoginseng saponins; (4) sinomenine.

**Figure S2. The gene primers for RT-qPCR in mouse liver tissue**

| Target gene |  | Sequence of primers (5′ to 3′) |
| --- | --- | --- |
| *GRP78*  *PERK*  *ATF4*  *DRP1*  *LC3*  *GAPDH* | Forward  Reverse  Forward  Reverse  Forward  Reverse  Forward  Reverse  Forward  Reverse  Forward  Reverse | GAAGGTTACCCATGCA AGCAATAGTTCCAGCGTCT  TGCGGCAACGCGTCCAGTAA  GCAGCGCCGGTTCATCCAGT  CCACAACATTGACCGAGATGA  ACCCATGAGGTTTCAAGTGC  CCACCTCTGTCGTGGCGCGTGGCCT  GCCACCGACTCACCACTAG  TTCTTCCTCCTGGTGAATGG  GTCTCCTGCGAGGCATAAAC  AACCCTTAAGAGGGATGCTGCCCTT  CTACGGGACGAGGAAACAC |


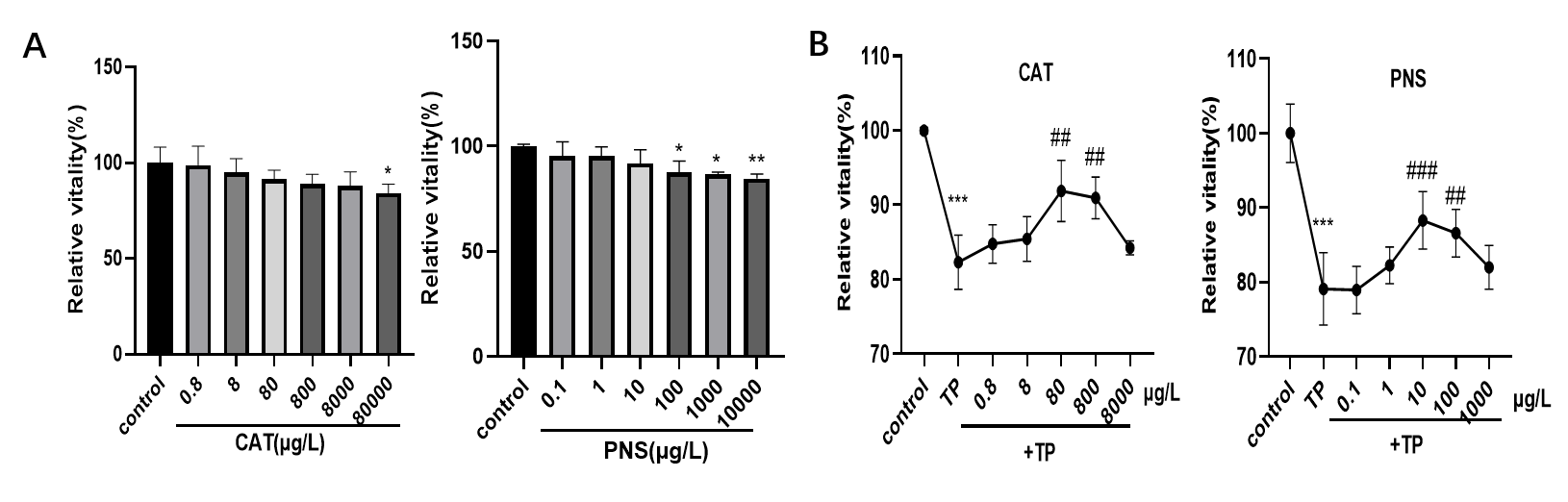


**Figure S3. The safe and protective dosage of CAT and PNS in HepaRG cells, respectively.**

A. The effect of 0.8-80000 μg/L CAT and 0.1-10000 μg/L PNS on the viability of HepaRG cells, respectively. B. 0.8-8000 μg/L CAT and 0.1-1000 μg/L PNS reversed the viability of HepaRG cells induced by TP (16μg/L), respectively. Compared with the control group, *^*^p<0.05, ^**^p<0.01, ^***^p<0.001*; compared with the TP group, *^#^p<0.05, ^##^p<0.01.*
